# [^18^F]‐NOTA‐FAPI‐04 PET/CT downgraded the staging of a breast cancer patient and changed their treatment management

**DOI:** 10.1002/pro6.1245

**Published:** 2024-11-27

**Authors:** Jingjie Qin, Jingjing Zhao, Jinming Yu, Yuchun Wei

**Affiliations:** ^1^ Department of Radiology Shandong Cancer Hospital and Institute Shandong First Medical University Shandong Academy of Medical Sciences Jinan Shandong Province China

**Keywords:** breast cancer, fibroblast activation protein, PET

## Abstract

A 47‐year‐old woman underwent [^18^F]‐FDG and [^18^F]‐NOTA‐FAPI‐04 PET/CT to assess the staging of suspected axillary lymph node enlargement following breast‐conserving surgery. The imaging with these two PET agents revealed starkly contrasting results. Significant [^18^F]‐FDG uptake in the right axillary fossa, intrathoracic muscles, and clavicle lymph nodes led nuclear medicine physicians to suspect metastasis. However, no uptake of [^18^F]‐NOTA‐FAPI‐04 was observed. Subsequently, the patient underwent an ultrasound‐guided biopsy of the enlarged axillary lymph nodes, which pathologically confirmed the diagnosis as inflammation. After a multidisciplinary discussion, the patient received radiotherapy for the right breast and 2.15Gy/F×28F for the tumor bed. She was discharged following the completion of her radiotherapy. Accurate diagnosis and staging are pivotal in selecting the optimal clinical treatment for breast cancer patients. Notably, [^18^F]‐NOTA‐FAPI‐04 PET/CT downgraded this patient's staging, significantly influencing the treatment strategy.

## Graphical abstract

In Brief: Qin et al. find an interesting case of a 47‐year‐old woman 41 days after breast‐conserving surgery and sentinel lymph node biopsy for right breast cancer. [^18^F]‐FDG PET/CT scan in the lymph nodes of right axillary fossa, right intrathoracic muscle, and right clavicle was considered as metastatic. However, no intense [^18^F]‐NOTA‐FAPI‐04 uptake was found on [^18^F]‐NOTA‐FAPI‐04 PET/CT scan. These [^18^F]‐NOTA‐FAPI‐04‐negative lymph nodes had been later confirmed by histopathology. Tumor staging was downgraded from cT1N3M0 to stage cT1N0M0.This interesting case suggested that [^18^F]‐NOTA‐FAPI‐04 PET may have the potential for differential diagnosis of benign and malignant lymph nodes and improving the accuracy of breast cancer staging.

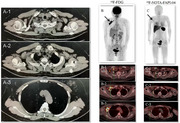

## 1 INTRODUCTION

^18^F‐fluorodeoxyglucose (^18^F‐FDG) PET/CT is pivotal in the staging, treatment, and prognosis of breast cancer. However, its sensitivity decreases with breast tumors smaller than 1 cm and lymph nodes.^1^, ^2^ Fibroblast Activating Protein (FAP), a type‐II transmembrane serine protease, is notably expressed in cancer‐associated fibroblasts (CAFs) within the stroma of epithelial carcinomas and tumor stroma.^3^, ^4^, ^5^ Radiolabeled FAP inhibitors (FAPI), such as Fludeoxyglucose (FDG), are emerging as promising radiotracers for malignancies.^6^ Specifically, ^68^Ga‐FAPI PET/CT excels in detecting primary breast masses, axillary lymph nodes, and distant metastases.^7^, ^8^ However, the short half‐life (68 minutes) and storage time of ^68^Ga limit its transportation over long distances. A novel alternative, aluminum‐[^18^F] fluoride (Al^18^F)‐labeled FAPI‐4 (FAPI‐04), referred to hereafter as [^18^F] AlF‐NOTA‐FAPI‐04,^9^, ^10^ is advantageous due to its longer half‐life (110 minutes).

Accurate diagnosis and staging are crucial for the optimal clinical management and prognosis of patients with breast cancer. This case involves a 47‐year‐old woman who, 41 days post‐breast‐conserving surgery and sentinel lymph node biopsy for right breast cancer, underwent a dual PET/CT examination with [^18^F]‐FDG and [^18^F]‐NOTA‐FAPI‐04. The imaging with these agents revealed starkly contrasting results: strong [^18^F]‐FDG uptake was observed in the right axillary fossa, intrathoracic muscles, and clavicle lymph nodes, leading physicians to suspect metastasis, whereas no [^18^F]‐NOTA‐FAPI‐04 uptake was detected. Histopathology later confirmed a downgrade in tumor staging from cT1N3M0 to cT1N0M0. Consequently, the patient underwent only right breast radiotherapy, thus avoiding additional surgeries, chemotherapy, and extensive radiotherapy.

## 2 CASE REPORT

The Ethics Committee of the Shandong Cancer Hospital and Institute, China approved this case study. Written informed consent for publication of the report was obtained from the patient.

**FIGURE 1:**
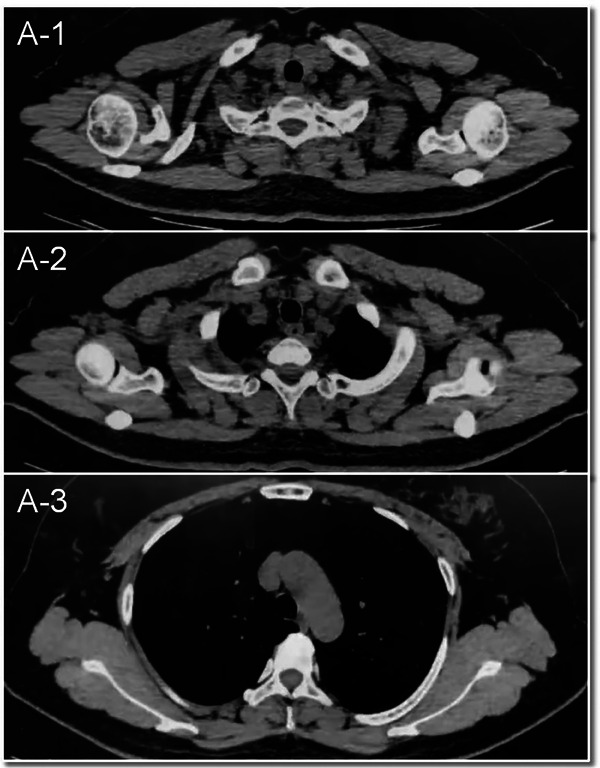
Preoperative plain CT scan.

**FIGURE 2:**
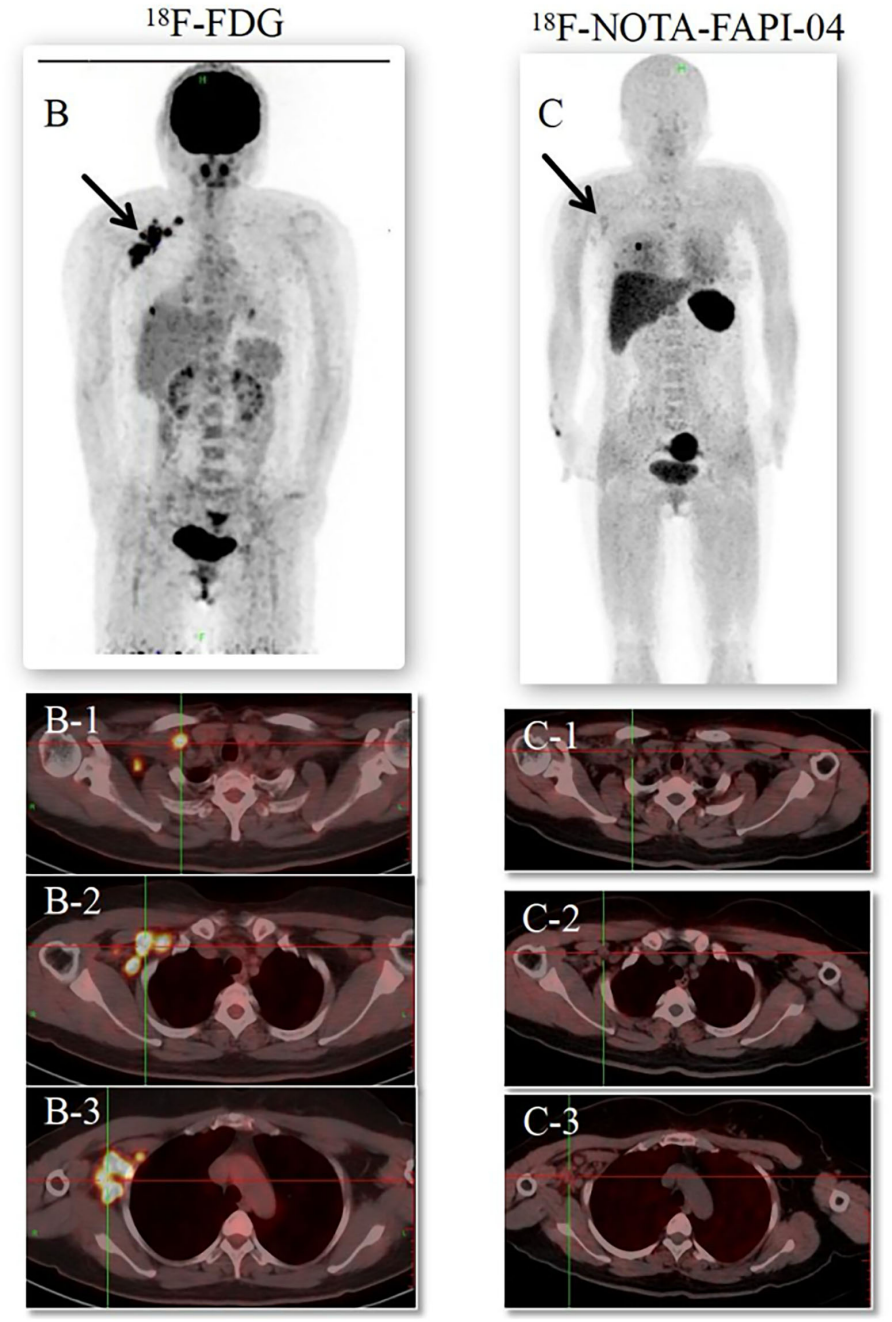
Comparison of [^18^F]‐FDG and [^18^F]‐NOTA‐FAPI‐04 PET/CT.

The subject of this study is a 47‐year‐old woman who was evaluated 41 days after undergoing breast‐conserving surgery and sentinel lymph node biopsy for right breast cancer. Preoperative CT scans revealed multiple lymph nodes of varying sizes in the right axilla (Figure 1). The postoperative pathological analysis revealed invasive breast cancer, non‐specific type grade II, totaling 6 points (3+2+1), with approximately 20% intraductal carcinoma. The tumor measured 1.4 cm× 1.2 cm× 1.2 cm, with no evident thrombus or nerve invasion. The surgical margins (inner, lower, outer, and upper) were cancer‐free. However, focal duct epithelial hyperplasia and atypical hyperplasia were noted in the frozen tissue from the outer and upper margins. No metastatic cancer was detected in any of the three examined lymph nodes (0/3) or the sentinel nodes (0/1 in each of three nodes). The final pathological staging was pT1cN0 (sn).’’ On palpation, multiple lymph nodes were detected in the right axilla, with the largest being approximately 2 cm in diameter. The patient subsequently underwent [^18^F]‐FDG PET/CT to assess the possibility of metastatic involvement of these axillary lymph nodes. The [^18^F]‐FDG PET/CT identified multiple enlarged lymph nodes in the right axillary fossa, right interthoracic muscle, and right clavicle, all showing significantly increased radioactivity uptake with a maximum SUV of 11.3 (Figure 2B). Radiologists considered these multiple lymph nodes to be metastatic. If metastatic, the patient would have required a second surgery, chemotherapy, and radiotherapy targeting the right breast, armpit, and ipsilateral supraclavicular lymph nodes. Subsequently, a [^18^F]‐NOTA‐FAPI‐04 PET/CT was performed for comparison. No intense uptake of [^18^F]‐NOTA‐FAPI‐04 was observed in the regions highlighted by the [^18^F]‐FDG PET (Figure 2C). These [^18^F]‐NOTA‐FAPI‐04‐negative lymph nodes, with a maximum SUV of 1.61, suggested the absence of nodal metastasis. This was later pathologically confirmed as inflammation via ultrasound‐guided biopsy of the enlarged axillary lymph nodes (Supplement 1). Consequently, tumor staging was downgraded from cT1N3M0 to cT1N0M0, and the patient underwent only right breast radiotherapy, thus avoiding secondary surgery, chemotherapy, and extensive radiotherapy.

The patient's prognosis is good. At regular follow‐up sessions following the completion of radiotherapy, the patient's clinical condition remained stable with no signs of recurrence or metastasis. We will include follow‐up information in a revised version of the article to better reflect the patient's long‐term prognosis

## 3 DISCUSSION

Fibroblast activating protein (FAP) is found on the surfaces of tumor stromal cells, macrophages, and tumor cells.^11^ It is highly expressed in over 90% of epithelial tumors,^12^ but remains low under physiological conditions.^13^ FAP actively supports the proliferation, migration, and invasion of tumors, endothelial cells, and immune cells, contributing to tumor invasiveness, neovascularization, and immune evasion.^14^, ^15^ Consequently, FAP represents a promising target and biomarker for anti‐tumor therapies. Currently, most FAPI PET imaging studies reported in the literature utilize 68Ga labeling, which presents several disadvantages: 1) A 68Ge/68Ga generator produces only 2–3 patient doses per elution, resulting in limited output; 2) its short half‐life (68 minutes) and brief storage period hinder long‐distance transportation; 3) high patient demand necessitates multiple generators, which are costly and pose challenges for radiation safety for operators. Conversely, 18F‐labeled FAPI‐04 is less commonly reported. Produced via cyclotron, ^18^F offers significant advantages over ^68^Ga, including: 1) a longer half‐life (110 minutes); 2) higher spatial resolution due to the positron energy of ^18^F; 3) greater popularity and utilization of accelerators in China, enhancing the feasibility of widespread adoption. Postoperative inflammatory responses can increase the metabolic activity of local tissues and lymph nodes, often leading to intense uptake in [^18^F]‐FDG PET/CT imaging of right axillary, pectoral, and clavicular lymph nodes. This can be a significant limitation of [^18^F]‐FDG PET/CT. This intriguing case highlights that [^18^F]‐NOTA‐FAPI‐04 PET/CT may offer potential for distinguishing between benign and malignant lymph nodes and enhancing the accuracy of breast cancer staging.

## CONFLICT OF INTEREST STATEMENT

No potential conflicts of interest were disclosed.

## ETHICS STATEMENT

This study was approved by the Ethics Committee of the Shandong Cancer Hospital and Institute (SDZLEC2021‐112‐02). An informed written consent was provided for the report publication.

## Supporting information



Supporting Information

## Data Availability

The data that support the findings of this study are available on request from the corresponding author. The data are not publicly available due to privacy or ethical restrictions.
