# Supplementary material for: [18F]‐NOTA‐FAPI‐04 PET/CT downgraded the staging of a breast cancer patient and changed their treatment management
Source: Precis Radiat Oncol. 2024 Nov 27;8(4):227–31. doi: 10.1002/pro6.1245 (PMC11934888; doi:10.1002/pro6.1245)
Supplement: Supplementary file 1 — Supporting Information [file PRO6-8-227-s001.docx]

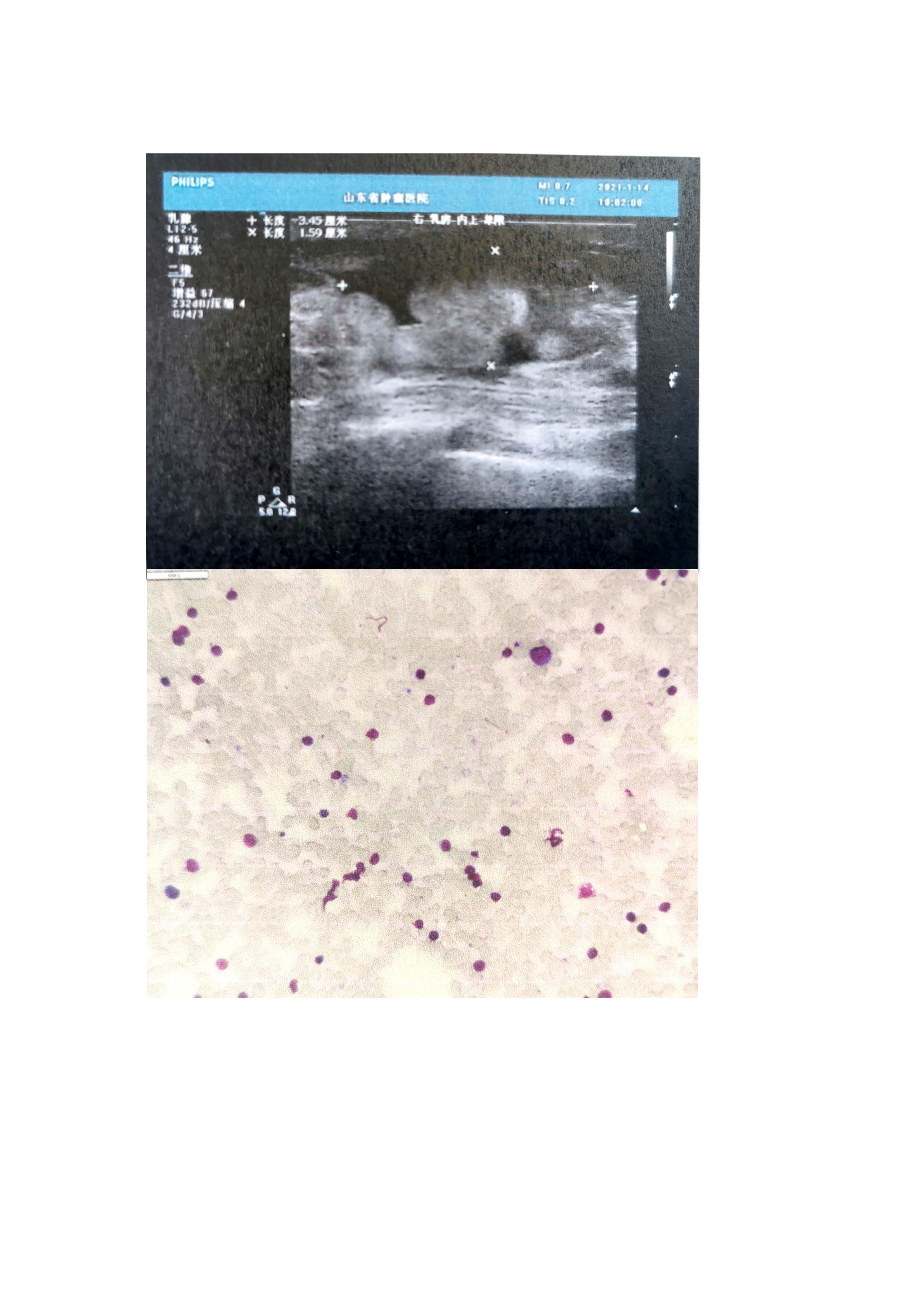


**Supplement 1** Ultrasound-guided biopsy of enlarged lymph nodes in the right axilla showed that no cancer cells were found.
